# Knowledge risk management in banks - An area for improving organizational performance

**DOI:** 10.1016/j.heliyon.2023.e22064

**Published:** 2023-11-04

**Authors:** Susanne Durst, Samuel Foli, Maura La Torre, Michele Borgia

**Affiliations:** a Department of Business Administration, Reykjavik University, Menntavegur 1, 102 Reykjavik, Iceland; b Department of Management and Business Administration, University “G. D'Annunzio” of Chieti-Pescara, Viale Pindaro, 42, 65127 Pescara, Italy

**Keywords:** Knowledge risk management, Knowledge management, Innovativeness, Agility, Responsiveness, Organizational performance, Banks, Replication study

## Abstract

Research on the topic of knowledge risks and their management in organizations is still very scarce, this also applies to empirical studies. However, to avoid the uncritical acceptance of empirical results, replication studies play a crucial role in science. Therefore, this study represents a replication study of the type of empirical generalization of the paper by Durst et al. (2019) which studied knowledge risk management (KRM) in private and public organizations. Considering the KRM and performance assumptions underlying the original study and the methodology used, the results at that time are reviewed using new data from 103 Italian cooperative banks. This paper contributes to the study of risks related to knowledge and its theoretical development by providing new empirical evidence from a different cultural, geographical and institutional context. Furthermore, it emphasizes the importance of replication studies for knowledge accumulation and theory development in management science.

## 1 Introduction

Recent societal and political developments have shown that a functioning risk management has become even more important [1,2]. Society as a whole and its actors are not only exposed to a multitude of internal and external risks, these risks are also interconnected [3,4]. Many of these risks are knowledge-based and given the importance of knowledge for the continuous and sustainable development of companies and thus societies, risk management should also address these types of risks in order to be better prepared for current and future challenges and opportunities [5]. Although knowledge is commonly seen as something positive and valuable, it cannot be overlooked that there are many moments in organizations when this picture is reversed, and knowledge becomes a negative risk factor. From this it can be concluded that in order to be able to understand knowledge and its importance for companies more holistically, not only its possible positive and negative side must be considered [6,7], but also risk management should be adapted or extended. Although the discussion of different knowledge risks, which have been defined “a measure of the probability and severity of adverse effects of any activities engaging or related somehow to knowledge that can affect the functioning of an organization on any level” [5, p. 2], has increased in recent years, the focus to date has been on the analysis of individual knowledge risks and their impact on different areas of the company. For example there are studies on knowledge loss [8,9], knowledge attrition [10], knowledge leakage [11,12], knowledge retention [13,14], knowledge hiding [15,16] or organizational forgetting [9,17]. Studies on knowledge risk management (KRM) are generally still rare and empirical studies even rarer [18,19]. This indicates that we are still dealing with a young field of research.

To further develop the topic and thus contribute to theory development, the aim of this work is to carry out a replication study of the work of Durst et al. [18] that investigated KRM in organizations. Replication studies help to demonstrate scientific integrity [20], this is an essential basis for the trust of researchers and society in research results and expertise [21]. As far as the role of trust is concerned, the pandemic and its course have clearly shown how dangerous the denial of scientific knowledge can be. Replications are considered crucial for increasing the credibility and practical usefulness of management research [22] and emerging fields of study [23]. This fits very well with the chosen topic, which is not only a management topic but also an emerging field of study. Despite the relevance, replication studies in social sciences were not considered for a long time [24,25] possibly due to an assumed lack of originality [26]. For example, van Witteloostuijn and van Hugten [27] who reviewed the current state of the art in social sciences by focusing on the statistical practices across top journals from a variety of disciplines found no paper that was based on replication. This situation, however, seems to be changing slowly [28] and the relevance of replications for continued progress of management science is increasingly voiced [22,26]. This paper follows this development and aims to contribute to further research progress and confidence in scientific work.

The choice of the Durst et al. [18] paper seems suitable for a replication study, since, to the authors' knowledge, it is the most comprehensive empirical study on KRM in private and public organizations to date. Additionally, the paper has been published in a very reputable journal, namely the Journal of Business Research and has been cited several times since its publication (149 times according to google scholar checked on October 13, 2023). In the present study, the original study is replicated with a different population, namely Italian cooperative banks. This type of replication is called empirical generalizations [28] and allows a test of the generalizability of the original study's findings regarding different contexts and thus to further fine-tune theory [25]. The authors of this paper believes that banks are well suited for the study of KRM, since banks are not only expected to have explicit risk management [29], but these organizations are also knowledge-intensive [30] and have recently come under pressure from the entry of financial technology (FinTech) organizations into the market [31].

Consequently, the aim of this paper is to examine the effect of KRM on organizational performance in Italian cooperative banks. More precisely and following the original study by Durst et al. [18], the paper examines the effect of KRM on “softer” measures of performance such as innovativeness, responsiveness, sustainability and agility. The present study makes an important contribution to theory development regarding KRM as the replication helps to determine the boundaries of the original study; it can be shown whether the earlier results are robust, generalizable and repeatable. Moreover, the replication helps to address the fragmentation and dispersion that is found in the area of risks related to knowledge and their management.

Against the background outlined before, the paper is structured as follows. Next, KRM and the main concepts are introduced, following Durst et al.*'s* study. In comparison to the original study, the relevant concepts are examined from the perspective of banks. Based on that we also develop a set of hypotheses. After that a description of the methodology follows, i.e., information on how the replication has been carried out at the methodological level. Subsequently, the results of the two studies are analyzed for similarities and differences. The paper ends with a conclusion section.

## 2 Literature review and development of hypotheses

In the following sections the relevant literature for this study and its aim (doing a replication study) is presented. It also includes the development of hypotheses.

### 2.1 Knowledge risk management in banks

Risk is inherent in the functioning of banks, which assume, manage and transform risks with the professionalism given by their “risk culture” [32]. Risk management, on the other hand, outlines the set of risk management processes and models through which banks implement their risk-based policies and practices, covering all the techniques and tools needed to monitor and control typical risks of banking activity, namely credit risk, market risk, interest risk, liquidity risk and operational risk [29]. Knowledge management (KM) also plays an important role in banks as it has a positive impact on their performance and value [33,34], on their ability to effectively manage innovation, especially technological innovation [35,36], and on their customer relation management [37,38].

When it comes to KRM, which has been defined as a set of tools and techniques for the identification, measurement and prevention and mitigation of the risks associated with the creation, application and conservation of organizational knowledge [18], the situation changes. KRM seems to be not yet known or less known in banks. La Torre [39] in her literature review on KRM in banks illustrates the scarcity of contributions in this research strand. Considering publications between 2001 and 2020, her review found only 12 articles dealing with issues related to KRM in banks or other financial firms. As underlined in the study by Durst et al. [18], research into knowledge risks in banks is also fragmented. Recent publications indicate that knowledge hiding has become a popular research topic. For example, Abubakar et al. [40], using artificial intelligence techniques, found that distributive, procedural and interactional (in)justice contributes to higher levels of knowledge hiding behaviors among bank employees using a sample of Turkish banks. While Alnaimi and Rjoub [41], using data collected from a sample of Jordanian commercial bank employees, investigated the link between perceived organizational support, psychological law, knowledge hiding behaviors and extra-role behavior, they also considered the mediation effect of knowledge hiding behaviors on such a relationship. This seems to be reflected in the general increase in interest in knowledge hiding in management literature (e.g., Anand et al., [42]; Nguyen et al. [43]).

Regarding other risks related to knowledge, van der Kleij et al. [44], for example, with reference to knowledge leakage, proposed the application of the Capability Opportunity Motivation-Behavior model to prevent data leakage, and Borgia et al. [45] analyzed the effects of technological knowledge risks on the relationship between work-life balance and job performance in banks. KRM in banks has been studied by Borgia and La Torre [46] who investigated the relationship between KRM and Organizational Change Management in local banks. In another study, these authors included KRM in an anti-money laundering training of bank personnel to propose a specific training evaluation model for banks engaged in the fight against money laundering [47].

### 2.2 Organizational performance

As it has been stressed in the literature (e.g., Becker and Gerhart, [48]; Richard et al. [49]), organizational performance is essential to survival and organizational success for all organizations; therefore, also for banks. Durst et al. [18] have focused in their study on more subjective measures of performance to underline that organizations today are looking not only at a broader number of types of performance but also at more diverse types in terms of measurability. This trend is also confirmed by the most recent research on the performance of banks and other financial companies. Several papers have focused on subjective measures of performance, such as studies on sustainability performance [[50], [51], [52]], job satisfaction [53,54], or leadership styles [55,56].

The following sections present the soft measures of performance used in the study following the original study. They are linked with the situations in banks.

#### 2.2.1 Innovativeness

It is commonly acknowledged that innovation matters [57], and this refers to all kinds of organizations [58] regardless of their operations and geographical location [59]. Consequently, innovativeness, which has been defined as “a continuous and systematic process, developed over time, and which focuses on the transformation of ideas into “successful reality” (Bessant and Tidd [60]). Innovativeness represents one of the key components for establishing an organization's technological readiness [61], in a crisis situation, this seems all the more important [62]. In relation to banks and other financial firms, several studies have also found a significant and strong relationship between the innovativeness of these organizations and their performance (e.g., Al-kalouti et al., [63]; YuSheng and Ibrahim, [64]; Rajapathirana and Hui [65]). The role of knowledge management [36,66] and risk management [67] have also been frequently considered in this regard. Other studies have analyzed the impact of bank innovativeness as a driver of social well-being [68] and of banks achieving a sustainable market position [69]. Shah and Khan [70] investigated the relationship between innovativeness and corporate social responsibility (CSR) in the retail banking sector of Pakistani banks and found that customers' CSR perceptions directly and positively influence banks' perceived innovativeness. Other studies have analyzed innovativeness through bank employees' behaviors, such as their innovativeness [71,72], autonomy [73] or work ethic [74].

Innovation activities are risky, thus in organizations showing too high levels of risk-taking the likelihood of failure also increases [75]. Compared to other sectors, banks are subject to stricter regulations and increased supervision to avoid/reduce excessive risk-taking [76]. Against this background, KRM appears to be particularly desirable for banks as it refers to a systematic way of applying tools and techniques to identify, analyze and respond to risks associated with the creation, application, and retention of organizational knowledge [18], that in turn could help banks in better handling the uncertainty associated with innovation and in this way also the avoidance of excessive risk taking. Therefore, the original hypothesis is also seen as relevant for banks.

H1 KRM positively impacts the innovativeness of banks.

#### 2.2.2 Responsiveness

The original paper used responsiveness as a further measure to determine the relationship between KMR and organizational performance. Responsiveness provides information on the extent to which a company reacts to market changes. Responsiveness is demonstrated through actions or the behavior of a system and relies on a range of capabilities to respond in a timely and appropriate manner to changes triggered by stimuli [77,78]. Recently published studies looked at the responsiveness of organizations in crisis situations, such as the Covid 19 pandemic, looking at different actions, behaviors and capabilities used in response to this crisis. Muneeb et al. [79], for example, examined responsiveness to the challenges of the Covid 19 pandemic based on the use of advanced ICT software such as Zoom, Google Meet and Microsoft Teams, while Rothschild et al. [80], in the context of human social care, demonstrated responsiveness to the pandemic crisis through training, safety and risk mitigation initiatives. Amis and Janz [81] looked at a human-centered approach to pandemic response and Athar [82] examined the role of organizational culture in responding to the post-Covid 19 crisis. The recent conflict between Russia and Ukraine further underscored the importance of organizational responsiveness, Balyuk and Fedyk [83] examined how US-American companies who decided to limit their presence in Russia in response to the operational and reputational effects of the war conflict.

Against this background and following the argumentation of the original study, in this study it is also assumed that the responsiveness of banks is influenced by the availability of recent and relevant knowledge. Hence, it is also argued that KRM can support this by providing necessary insight into positive and negative risks related to extant knowledge sources and their impact on the banks’ responsiveness. Thus, the original hypothesis was kept and transferred to banks.

H2 KRM positively impacts the responsiveness of banks.

#### 2.2.3 Agility

The original study used agility as a further soft performance measure. Agility describes the organizational ability of companies not only to recognize competitive opportunities and threats, but also to respond to them [84]. In doing so, these companies draw on past knowledge and combine it with current experience [85]. Considering banking and financial sectors, agility has been studied with particular attention to the role of banks' information technology capabilities [[86], [87], [88]] and alignment [89,90]. In other studies, the organizational agility of banks was measured applying specific methods and models, Barati et al. [91] using the data envelopment analysis (DEA) approach, Mirsepaci and Farshchi [92] employing the hexagonal model for the agility in the public sectors, Arefnezhad et al. [93] proposing a model for the implementation of organizational agility based on the flexibility of bank's human resources, while in Zinkanlou [94], an organizational resilience model based on organizational agility was designed for Sepah bank branches. Aburub [95] investigated the impact of enterprise resource planning (ERP) on agility in the banking sector in the Middle East, in which a new model was tested. In other research, organizational agility of banks was related to the quality of working life [96], to organizational performance [97,98], to leadership practices and the organizational commitment of employees [99,100]. Banks' organizational agility was also considered as a moderating or mediating variable [101,102].

Following the argumentation of the original study that risk management not only serves to reduce or even avoid negative risks, but also to maximize the success of opportunities, it can be concluded that agile banks should also benefit from KRM. Consequently, the original hypothesis is followed.

H3 KRM positively impacts the agility of banks.

#### 2.2.4 Organizational success

Organizational success is an integrative term encompassing various aspects of organizational functioning. According to Flamholtz and Aksehirli [103], organizational success can be the outcome of an interplay of different aspects, for example, identification and definition of a solid market niche; development of products or services for the selected market niche; acquisition and development of resources necessary to run the firm; development of day-to-day operational systems; development of the management systems necessary for the long-term functioning of the organization; and finally, development of the organizational culture crucial to guide the firm. Against the relevance of organizational success for organizations and their long-term survival, the measurement of success is crucial even though it is challenging [104]. Organizational success can be measured in a variety of ways, depending for example on the sector or stage of development. To measure organizational success in comparison with other similar entities is justified as well. With reference to the banking industry, Chen [105] identified several critical factors that determine the bank's success, including a good reputation and image, effective asset and liability management, and the ability to develop new business to satisfy unsatisfied demand. In the study by Artamonov et al. [106], involvement of staff is considered as a fundamental factor for the success of modern banks. Monitoring, coordination, design, training, and institutional environment were identified by Ika et al. [107] as a critical success factor for 10.13039/100004421World Bank projects, while the results of another research showed privacy and security, customer support, convenience and efficiency to be key factors for the success of sustainable mobile banking [108]. Maina [109] identified the success factors of commercial banks in Kenya, such as mainly employment of modem technology, reasonable cost of services and sound corporate governance. In another research, the key success factors affecting Islamic and conventional banks were explored, making a comparison before and during the COVID-19 pandemic period [110]. Risk management can help organizations not only to minimize their risks, but also to maximize their chances of success [111]. The ISO 31000:2018 highlights the role of risk management for improving performance, encouraging innovation, and supporting the achievement of objectives. Consequently, it can be expected that KRM in banks can contribute to achieving organizational success. Therefore, the original hypothesis is followed.

H4 KRM positively impacts the success of banks.

#### 2.2.5 Organizational sustainability

As has been stressed in the original study, the topic of organizational sustainability is on the agenda of a growing number of organizations. This also applies to financial institutions, which also need to be more environmentally and socially responsible, as it is less and less possible to consider them responsible when they finance all companies regardless of their business practices [112]; thus highlighting the three main pillars of sustainability - environment, social responsibility and economy. In a recent literature review [113], it was highlighted that, despite the fact that the banking sector can impact sustainable development directly (to a lesser extent) and indirectly (to a greater extent), research on the sustainability of banks and other financial companies is still in a rather initial phase, and lacks contributions, in particular, on the concept of sustainable banking and its measurement [113]. In this regard, the authors of the above-mentioned review found sixty-three papers on bank sustainability and its measurement, starting from 1983 until 2019, almost all proposing qualitative case studies [113]. After 2019, several studies have focused on the organizational sustainability of Islamic banks [114,115]. In Ecer and Pamucar [51], bank sustainability performance was measured using a novel multi-criteria framework. Other research dealt with the relationships between sustainability performance of banks and audit committee characteristics [116], knowledge sharing [117], FinTech adoption [118], Green Banking Practices and Employee Green Behavior, and green finance [119,120], as well as gender diversity [121]. Moreover, the factors that can influence the sustainability of neobanks [122], and the quality of banking sustainability reporting [123,124] have been studied.

Against the background of the role of knowledge for the organizational sustainability of banks as well, it can be concluded that they should also benefit from KRM to achieve a balance between risk-taking and possible consequences. The original hypothesis is thus followed.

H5 KRM positively impacts the organizational sustainability of banks.

**Fig. 1:**
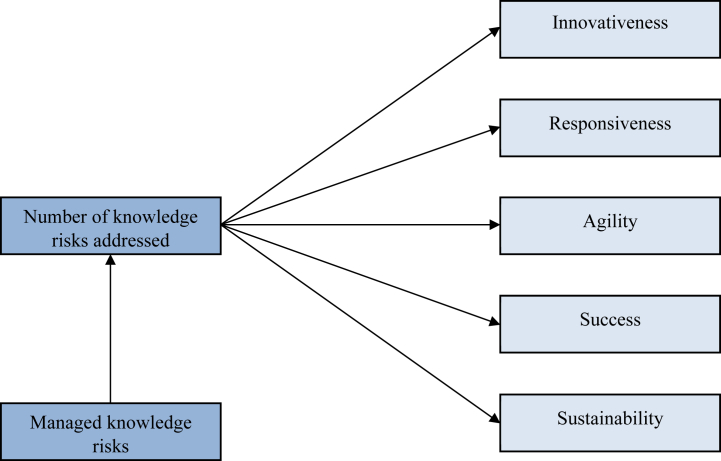
Theoretical framework.

Fig. 1 depicts the framework of the study.

## 3 Methodology

As mentioned before, this study replicated the study by Durst et al. [18] using empirical generalizations as the type of replication [28]. Thus, the original study was tested in a different context. In the present case, to a different cultural, geographical (Italy) and institutional context (cooperative banks). Mueller-Langer et al. [125] called it a scientific replication as different data are used compared to the original study. Otherwise, the two assumptions of Tsang and Kwan [25] regarding replication were followed, namely (1) the authors used the same research methods as used in the original study, and (2) the replication was carried out at a time after the original one had been completed. In case of the type of empirical generalization, research recommends that the research procedures of the original study are closely followed [25] as the findings of the new study are affected by what is changed from the original study. However, as this is very difficult and depends very much on the available information, Singh et al. [23] talk in this context about a good-enough replication which is a study that follows as closely as possible the described methodology of the original study. In case variations are made they should be justified. The following sections contain information on how this replication study proceeded.

### 3.1 Sample and data collection

In contrast to the study by Durst et al. [18], which did not distinguish between organizations and sectors, this study focused on one sector, namely banks and in particular cooperative banks. In addition, the original study did not distinguish between individual countries, whereas this study focused on a single country, Italy. Banks are an interesting subject for KRM research and thus for a replication study, as they are knowledge-intensive organizations that need to manage risk and have recently been challenged by new entrants that have put their business models and underlying knowledge to the test. Hence, banks are also exposed to several human, technological and operational knowledge risks in both day-to-day and extraordinary operations, such as the Italian CCBs, which following a reform of the cooperative credit system, had to join two Cooperative Banking Groups on a national scale [126,127].

Data collection occurred between July and October 2022 in the form of an online questionnaire administered to a sample of Italian cooperative credit banks (CCBs). Access to the banks was made possible by approaching local cooperative credit associations to which one of the authors of this study has access. These associations then also arranged for the online link to be sent to the respective CCBs. The questionnaire used covered exactly the questions of the original study, with the only difference that the wording was adapted to the banks. Like the original study, the questionnaire used in this study was also pre-tested. The questionnaire was pre-tested with one professor familiar with the topic under investigation and two bank employees of the target population. As the study did not include vulnerable people, no ethics approval was required. Participants gave their informed consent by conducting the study, i.e., by starting (completing) the online questionnaire. To be able to do this, potential participants were informed about the study and the aim of the study. Furthermore, information about data use and anonymity of the collected data was provided.

A total of 103 fully completed questionnaires were collected from managers and employees of Italian CCBs. At the time of the study, there were 228 of these banks that were familiar with the issues in question.

Even though this study is limited to one population, namely Italian cooperative banks, the use of a self-reported survey may still raise questions about common method variance (CMV). As a result, Harman's single-factor test was conducted (the same test was applied in the original study) which revealed that only 44.7 % of variance could be explained by a single factor, which is below the recommended threshold of 50 %. Hence, CMV does not appear to be a problem in this replication study.

### 3.2 Measures

In this replication study, the same constructs were used as in the original study. Consequently, six constructs were studied, namely KRM, innovativeness, agility, responsiveness, sustainability and success; the same scales were used. The same refers to the binary question used to determine if respondents incorporate knowledge risks into their risk management strategies. Furthermore, and as in the original study, a total of 16 knowledge risks were covered, which were also treated as items and used as a regression component in the estimation of KRM using maximum likelihood methods. Organizational performance was measured using the subjective self-report measures proposed in the original study, where respondents were asked to rate their company's innovativeness, agility, responsiveness, sustainability and success compared to competitors on a 5-point Likert scale.

It should be mentioned that no control variables were used in the replication study. This decision was made in view of the nature of the sample. Since the present study focuses exclusively on Italian cooperative credit banks, there is a sample that has a high degree of homogeneity, which reduces the likelihood of confounding effects.

### 3.3 Statistical method

The statistical methods used in this replication study also followed the original methods as closely as possible. That means, first, a descriptive statistical analysis was carried out using Microsoft Excel to estimate means, standard deviations, and correlations (please refer to Table 1, Table 2). Next, as the original study did, structural equation modeling (SEM) technique was employed to conduct inferential statistical analysis of the formulated hypotheses. STATA software version 17 was used to conduct this analysis. As in the original study, the evaluation of model fitness was performed according to the guidelines proposed by Hu and Bentler [128], which included the use of a multi-index presentation format that incorporated the Standardized Root Mean Squared Residual (SRMR), the Tucker-Lewis Index (TLI), the Comparative Fit Index (CFI), and the Root Mean Square Error of Approximation (RMSEA). Ultimately, the model's robustness was determined based on established thresholds for the fit indices (please refer to Table 3).

**Table 1:** Comparison of means and standard deviation.

Variable	Mean	S.D.
1. Manage knowledge risks	0.82 (1.13)	0.04 (1.14)
2. Number of knowledge risks addressed	0.52 (3.82)	0.03 (4.17)
3. Success	3.93 (4.39)	0.10 (1.66)
4. Responsiveness	3.82 (4.10)	0.09 (1.43)
5. Innovativeness	3.64 (4.19)	0.09 (1.79)
6. Sustainability	3.68 (3.93)	0.09 (1.73)
7. Agility	3.92 (3.96)	0.09 (1.85)

Note: The findings of Durst et al. (2019) are in parenthesis.

**Table 2:** Comparison of correlation coefficients.

Variable	1	2	3	4	5	6	7
1. Manage knowledge risks	1.00						
2. Number of knowledge risks addressed	.313** (.281**)	1.00					
3. Success	.219* (−0.006)	.304**(0.277**)	1.00				
4. Responsiveness	.305** (−0.077)	.308**(0.141)	.782**(0.376**)	1.00			
5. Innovativeness	.326** (−0.062)	.213*(0.336**)	.653**(0.607**)	.723**(0.435**)	1.00		
6. Sustainability	.271** (−0.075)	.221*(0.279**)	.603**(0.560**)	.640**(0.478**)	.595**(0.712**)	1.00	
7. Agility	.277** (−0.610)	.247*(0.337**)	.608**(0.587**)	.644**(0.571**)	.668**(0.571**)	.673**(0.780**)	1.00

Note: The findings of Durst et al. (2019) are in parenthesis; correlation coefficient is significant at * p < .05 (two-tailed), **p < .01 (two-tailed).

**Table 3:** Summary of hypotheses tests.

Variable	Durst et al. (2019)^α^	Replication study^β^
Innovativeness	.34**	.21*
Responsiveness	.14	.29**
Agility	.34**	.28**
Success	.30**	.30**
Sustainability	.28**	.24*
Number of knowledge risks addressed	.28**	.32**
Sample size (n)	179	103
Model fit	χ^2^ = 29,716, *df* = 21, SRMR = .057; RMSEA = .048, CFI = .987, TLI = .972	χ^2^ = 12,039, *df* = 10, SRMR = .075; RMSEA = .045, CFI = .994, TLI = .985

First, the results of this replication study indicate that all model fit indices indicate good model fit (SRMR <0.08; RMSEA <0.06; CFI >0.95; TLI >0.95; chi-square with non-significant test statistic); comparable with the original study.

## 4 Results

In this section, the results are presented and compared with those of the original study. To facilitate the comparison between the present replication study and the work of Durst et al. [18], the descriptive statistics of both studies are presented; including means and standard deviations in Table 1 and the correlation in Table 2. Microsoft Excel was utilized for these analyses due to its convenience, especially for basic descriptive analysis, immediately following data cleaning.

The results of Table 1 show, on the one hand, that the participants in the original study rated the importance of the relationship between KRM and the performance indicators higher (please refer to the means, all are higher) and on the other hand, as expected, that the standard deviation (S.D.) is lower in a homogeneous sample (in the replication study: banks from one country) than in a heterogeneous one (original study: private and public organizations representing different sectors).

As can be seen from Table 2, the findings of this study indicate a significant positive correlation between KRM and the number of knowledge risks addressed by Italian banks (r = 0.313, p < .01). This is consistent with those reported by Durst et al. (2019) (significant at p < .01 and showing a positive direction as well). In addition, the replication study reveals a significant positive correlation between the number of knowledge risks addressed in the banks’ KRM and all organizational performance measures. This finding is somewhat consistent with the original study. This means that the replication study also shows that including a variety of different knowledge risks leads to better results for different types of business performance. The present study also shows consistency with the original study in that all types of organizational performance have significant positive relationships with each other, as indicated by correlation coefficients ranging from 0.595 to 0.782 (p < .01).

The authors proceeded in a similar manner. STATA software was used to test the proposed hypotheses using SEM, as it offers SEM capabilities and functions not available in Microsoft Excel. Table 3 shows the SEM results of the replication study and the original study.

Turning to the hypotheses, the authors observed that in both the current (replication) study and Durst et al.'s [18] work, KRM demonstrates a robust positive influence on both agility and organizational success. Thus, H1 and H5 can be confirmed with a relatively high degree of consistency across the two studies. To discover and discuss any differences in the results of the two studies, the authors first looked at how KRM affects innovativeness. In the study by Durst et al. [18], KRM showed a strong positive effect on the innovativeness of the organizations studied (r = 0.34, p < .01), this effect was only partially confirmed (r = 0.21, p < .05) in this study. One possible explanation for this result could be related to the fact that banks, especially cooperative banks, are not only subject to stricter regulations, but are also more conservative when it comes to developing new products, services, etc. In contrast, the study by Durst et al. [18] did not distinguish between sectors or organizations. Another slight difference in the results of the two studies concerns the effect of KRM on organizational sustainability. While Durst et al.'s study found a strong positive impact of KRM on organizational sustainability (r = 0.28, p < .01), the present study only demonstrated a partial effect (r = 0.24, p < .05). In this respect, the hypotheses H3 and H4 are only partially supported by the results.

The most notable difference between the original study and this study was found in the link between KRM and responsiveness. The results obtained vary considerably. According to the original study, KRM showed no statistical evidence of impact on responsiveness (r = 0.14, p ≥ .05), while in this study, a robust statistical impact is observed (r = 0.29, p < .01). This difference can possibly be explained by the different data collection periods of both studies, the data of the study by Durst et *al.* [18] were collected before the Covid-19 pandemic, while data collection for this study took place in the post-Covid era. The pandemic required rapid responses to the situation by adopting new ways of working and servicing clients at short notice, and the CCBs may have been better equipped to meet the challenges of the pandemic at the time the data was collected. This may have resulted in a more pronounced or heightened level of responsiveness compared to the results observed in the original study. Thus, the results give a strong justification to confirm H2. Table 4 summarizes the hypotheses tests of both studies.

**Table 4:** Summary of hypotheses tests.

Hypotheses	Durst et al. (2019)	Replication study
H1: KRM positively impacts the success of an organization	✔	✔
H2: KRM positively impacts the responsiveness of an organization	**×**	✔
H3: KRM positively impacts the innovativeness of an organization	✔	**∼**
H4: KRM positively impacts the sustainability of an organization	✔	**∼**
H5: KRM positively impacts the agility of an organization	✔	✔

Note: ✔ = confirmed; **∼** = partially confirmed; **×** = rejected.

## 5 Discussion

The perception of knowledge has recently been questioned, both theoretically and empirically in the knowledge management (KM) literature. Following this new line of inquiry, which highlights possible risks related to knowledge, the authors of this paper did a replication study on a study by Durst et al. [18] who studied the relationship between KRM and subjective measures of organizational performance both in private and public organizations. Specifically, this relationship was examined in Italian cooperative credit banks. Overall, the authors find support for the relationship between KRM and success, responsiveness and agility. Partial support was found for the relationship between KRM and innovativeness and sustainability. Compared to the original study, this study finds a statistically significant contrast regarding the relationship between KRM and responsiveness. The relationships between KRM and innovativeness and sustainability were confirmed in the original study but not in the replication study. As it was noted before, the discrepancy between the findings and those of the original study can be explained first by the different times at which the data were collected and second by the different populations involved in the studies. The pandemic indeed required all types of organizations to identify and implement quick responses to keep their operations going [129]. Against this backdrop, and the fact that the pandemic was already on the downswing at the time of data collection, it can be assumed that CCBs have not only acquired new capabilities to better respond to external crisis situations [130], but that they have also adapted their (K)RM accordingly [131], which is reflected in a positive and significant relationship between KRM and bank responsiveness.

This replication study is based on the type of empirical generalization which means a past study was repeated on a different population, so the authors could find out how far the results of the original study are generalizable to other populations. According to the authors’ knowledge, this study is the first replication of the study by Durst et al. [18] and the hypotheses used at that time were put to the test. Despite the discrepancies, it can be said that the main message of the original study, i.e., that there is a strong positive correlation between KRM and different types of organizational performance, could also be underlined in this study. Consequently, we as researchers can make a prediction, i.e., the theory is in existence before the data. The authors think that this result is very important for this emerging field of research as it increases the credibility of the research activities and thus also the possibility that the recommendations for practice derived from these are based on a sound and valid approach.

## 6 Conclusions

In this study, a replication, type empirical generalization, of the study by Durst et al. [18] was carried out in order to find out to what extent the original results are transferable to other populations, in this case to Italian cooperative banks; that is, to what extent the results of that time are generalizable. Despite slight variances between the studies which are not surprising, the results of both studies go in a similar direction - there is a strong and positive correlation between KRM and organizational performance. This clearly indicates that the result of the relationship between KRM and performance measures at that time was not only robust but also generalizable. The results also suggest that the methodology used at the time can be replicated. The level of knowledge thus achieved forms a valid basis for further research on KRM. Recommendations for managerial practice can thus be made with greater confidence. Hence, it can be concluded that an extension and improvement of the theory of KRM in organizations was possible. The variances found help strengthen the current theory. More precisely, the findings presented in this study suggest that KRM research would clearly benefit from an inclusion of the aspect of environmental change (i.e., the temporal context), especially when examining the relationship between KRM and organizational performance, to avoid comparing so-called apples with oranges. This means that the original theory would have to be adapted in terms of the role of context; given the state of development of the state of research, it is not surprising that this has not yet been done. In this context, studies that collect data at different points in time would also be useful to see if and how different types of organizations change their behavior regarding KRM and what effect this has on the organizations' performance. The authors believe that the present study has made an important contribution to theory development and hope that future research will start from here when developing KRM theory further. Despite this promising result, possible limitations of this work should be emphasized. As far as the conduct of the replication study is concerned, the authors tried to come as close as possible to the original study, as described before, but a hundred per cent replication of a study is never possible and must be acknowledged. Like the original study, this study was based on a cross-sectional approach. Changes over time could therefore not be controlled but appear relevant in the context of risks and their management.

In summary, the authors of this paper hope that they could show that doing a replication study is a meaningful and needed type of research as it can facilitate knowledge accumulation and theory development in KRM and KM in general. Regarding the latter, this paper responds to a relatively old call by Singh et al. [23] who stressed that the field of knowledge management would benefit from replication studies that are dedicated to building theoretical understanding and empirical generalizations. The authors thus invite researchers in the field of knowledge management, which includes KRM, to respond to this and contribute more to knowledge accumulation and theory development; different types of replication studies, such as re-analysis of data or conceptual extension, on seminal KM papers could help or make the beginning. This focus would also help the field to lead and advance stronger academic discussion that is based on solid empirical evidence, which in turn makes an important contribution to continued science progress in management research.

## Data availability statement

The data associated with this study has not been deposited into a publicly available repository. The data will be made available on request.

## CRediT authorship contribution statement

**Susanne Durst:** Writing – review & editing, Writing – original draft, Validation, Supervision, Project administration, Methodology, Data curation, Conceptualization. **Samuel Foli:** Writing – review & editing, Writing – original draft, Visualization, Formal analysis, Data curation. **Maura La Torre:** Writing – original draft, Investigation, Writing – review & editing. **Michele Borgia:** Resources, Investigation.

## Declaration of competing interest

The authors declare that they have no known competing financial interests or personal relationships that could have appeared to influence the work reported in this paper.
