# Supplementary material for: Knowledge risk management in banks - An area for improving organizational performance
Source: Heliyon. 2023 Nov 4;9(11):e22064. doi: 10.1016/j.heliyon.2023.e22064 (PMC10663912; doi:10.1016/j.heliyon.2023.e22064)
Supplement: Multimedia component 1 [file mmc1.docx]

**Questionnaire on knowledge risks Cooperative Credit Banks**

**1. General questions related to the risk management of knowledge**

1. Does the organization do (knowledge) risk management?

Yes

No

I don’t know

1. Which knowledge risks are considered in your risk management?

| **Items** | **Yes** | **No** | **Don’t know** |
| --- | --- | --- | --- |
| Knowledge loss |  |  |  |
| Knowledge leakage |  |  |  |
| Knowledge spill over |  |  |  |
| Risks related to knowledge gaps |  |  |  |
| Relational risks |  |  |  |
| Risk of using disinformation or unreliable information |  |  |  |
| Risk of improper application of knowledge |  |  |  |
| Unlearning |  |  |  |
| Forgetting |  |  |  |
| Knowledge waste |  |  |  |
| Knowledge hiding |  |  |  |
| Knowledge hoarding |  |  |  |
| Risks related to social media |  |  |  |
| Risks related to cyber-crime |  |  |  |
| Risks related to digitalization |  |  |  |
| Others, please specify_____ |  |  |  |

**2. Questions related to the performance of the organization**

(Measure on Likert scale: 1 being “extremely disagree” 2 “disagree,” 3 “neutral” 4 “agree” and 5 “extremely agree”)

Compared with other comparable cooperatives, my organization

…………… is more successful.

…………… is more innovative.

…………… is more sustainable, i.e., it focuses on meeting the needs of the present without compromising the ability of future generations to meet their needs.

…………… has a better responsiveness to changes in the environment, e.g., in terms of an external crisis

…………… is more agile
